# Preparation of mouse pancreatic tumor for single-cell RNA sequencing and analysis of the data

**DOI:** 10.1016/j.xpro.2021.100989

**Published:** 2021-12-04

**Authors:** Aizhan Surumbayeva, Michael Kotliar, Linara Gabitova-Cornell, Andrey Kartashov, Suraj Peri, Nathan Salomonis, Artem Barski, Igor Astsaturov

**Affiliations:** 1 The Marvin & Concetta Greenberg Pancreatic Cancer Institute, Fox Chase Cancer Center, Philadelphia, PA 19111, USA; 2 Division of Allergy and Immunology, University of Cincinnati, Cincinnati, OH 45221, USA; 3 Division of Human Genetics, University of Cincinnati, Cincinnati, OH 45221, USA; 4 Division of Biomedical Informatics, University of Cincinnati, Cincinnati, OH 45221, USA; 5 Cincinnati Children’s Hospital Medical Center and Department of Pediatrics, University of Cincinnati, Cincinnati, OH 45221, USA; 6 Datirium, LLC, Cincinnati, OH 45226, USA; 7 Biostatistics and Bioinformatics Facility, Fox Chase Cancer Center, Philadelphia, PA 19111, USA; 8 Cancer Biology Program, Fox Chase Cancer Center, Philadelphia, PA 19111, USA

**Keywords:** Bioinformatics, Sequence analysis, Cell Biology, Cell isolation, Single Cell, Cancer, RNAseq, Molecular Biology

## Abstract

Preparation of single-cell suspension from primary tumor tissue can provide a valuable resource for functional, genetic, proteomic, and tumor microenvironment studies. Here, we describe an effective protocol for mouse pancreatic tumor dissociation with further processing of tumor suspension for single-cell RNA sequencing analysis of cellular populations. We further provide an outline of the bioinformatics processing of the data and clustering of heterogeneous cellular populations comprising pancreatic tumors using Common Workflow Language (CWL) pipelines within user-friendly Scientific Data Analysis Platform (https://SciDAP.com).

For complete details on the use and execution of this protocol, please refer to Gabitova-Cornell et al. (2020).

## Highlights

• Isolation of single-cell suspension from pancreatic tumor
• Step-by-step guidance to the generation of quality single-cell RNA libraries
• An online informatics pipeline SciDAP for downstream processes
• SciDAP uses open source pipelines to obtain reproducible results

## Graphical abstract

**Figure:**
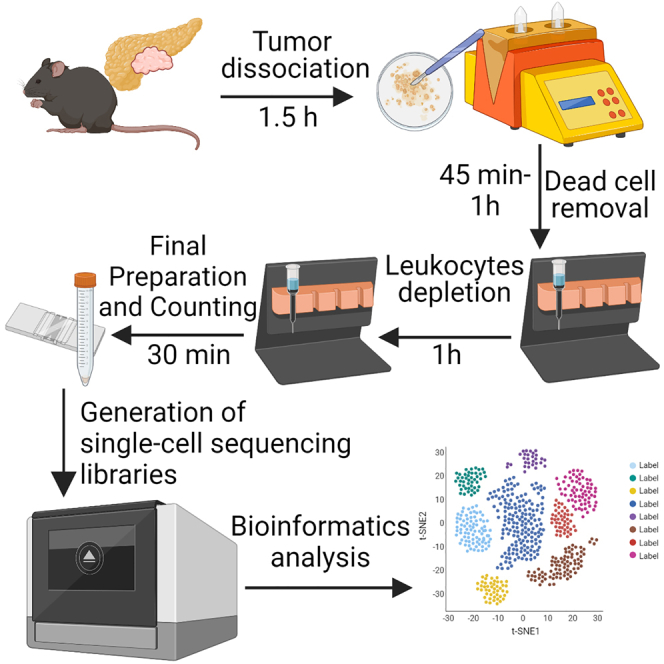

## Before you begin

1. Prepare reagents Enzyme D, Enzyme R, Enzyme A by reconstitution of the lyophilized powders from the Tumor Dissociation Kit(mouse) according to Table 1. Make aliquots (100 μL of Enzyme D, 50 μL of Enzyme R, and 12.5 μL of Enzyme A per sample) and store them at −20°C, to avoid repeated freeze-thaw cycles. These solutions are stable for 6 months after reconstitution.

**Table 1:** Media and reagents

Enzyme	Reconstitution solution	Volume	Notes
D	RPMI 1640 or DMEM	3 mL	filter (0.22 μm) prior to aliquoting
R	RPMI 1640 or DMEM	2.7 mL	
A	Buffer A	1 mL	do not vortex
2. Prepare sterile 1**×** PBS (Ca2+ and Mg2+ free). Pre-cool PBS at +4°C.
3. Prepare sterile, double-distilled water.
4. Pre-cool the centrifuge to +4°C.
5. Prepare the necessary stock solutions and sterile tools according to the materials and equipment section.
6. Prepare 1**×** Binding Buffer from 20**×** Binding Buffer Stock Solution supplied with the Dead Cell Removal Kit by diluting in double-distilled water.

**CRITICAL:** Do not use deionized water for dilution.

**CRITICAL:** Binding of Dead Cell Removal MicroBeads requires Ca2+. The presence of the ion chelator EDTA will abolish binding. 1**×** Binding Buffer is optimized for best Dead Cell Removal MicroBeads binding. The use of a different buffer may lead to poor dead cell removal efficiency.

**CRITICAL:** Mouse primary tumor cells carry potentially hazardous pathogens. Procedures should be performed under BSL-2 conditions.

Tumor-bearing mice were observed twice weekly until signs of sickness appeared or animals showed distress or weight loss of more than 10%, per the local Institutional Animal Care and Use Committee (IACUC) guidelines.

## Key resources table

**Table:** 

REAGENT or RESOURCE	SOURCE		IDENTIFIER
**Chemicals, peptides, and recombinant proteins**			

Dulbecco's phosphate-buffered saline (DPBS) (Ca2+and Mg2+ free)	Sigma-Aldrich		D5652
Dulbecco's Modified Eagle Medium (DMEM)	Corning		50-013-PB
Fetal Bovine Serum (FBS)	HyClone		AE28209281
Bovine Serum Albumin (BSA)	Fisher Scientific		BP1600-100
Penicillin/Streptomycin	Cellgro		MT30-002-CL
EDTA	Fisher Scientific		BP120-500
Trypan Blue Stain	Fisher Scientific		15250061

**Critical commercial assays**			

Tumor Dissociation Kit, mouse	Miltenyi Biotec		130-096-730
Dead Cell Removal Kit	Miltenyi Biotec		130-090-101
CD45 MicroBeads, mouse	Miltenyi Biotec		130-052-301
Red Blood Cell Lysis Solution	Miltenyi Biotec		130-094-183
Annexin V MicroBead Kit	Miltenyi Biotec		130-092-052
Chromium Single Cell 3′ Library, Gel Bead & Multiplex Kit and Chip Kit V3	10X Genomics		PN-1000092
Bioanalyzer High Sensitivity DNA Kit	Agilent Technologies		5067–4626
Qubit dsDNA HS Assay Kit	Thermo Fisher Scientific		Q32851

**Deposited data**			

Single-cell RNAseq data	Sequence Read Archive (SRA), https://www.ncbi.nlm.nih.gov/sra		PRJNA657051

**Experimental models: Organisms/strains**			

Male and female mice of KPC genotype (*LSL-Kras*^*G12D*^*;Trp53*^*f/f*^*; Pdx1*-*Cre*) were generated in house. Original strains were obtained from JAX.org. Tumor-bearing mice at age of 7–8 weeks were used for tumor harvesting	The Jackson Laboratory, Bar Harbor, ME		JAX 014647; 019104; 008462

**Software and algorithms**			

CellRanger 4.0.0	10X Genomics	https://support.10xgenomics.com/	
Seurat version 4.0.1	New York Genome Center	https://satijalab.org/seurat/install.html	
CWL pipelines for scRNA-Seq data processing	Datirium, LLC	https://github.com/datirium/workflows	

**Other**			

gentleMACS™ Dissociator	Miltenyi Biotec.	130-093-235	
gentleMACS C Tubes	Miltenyi Biotec.	130-093-237	
MACS MultiStand separator	Miltenyi Biotec.	130-042-303	
MACS Separation Columns (LS)	Miltenyi Biotec.	130-042-401	
MACSmix™ Tube Rotator	Miltenyi Biotec.	130-090-753	
37°C incubator	N/A	N/A	
FlowmiTM Tip Strainers 70 μm	SP BEL-ART	H13680-0070	
50 mL sterile falcon tubes	CellStar	227261	
15 mL sterile falcon tubes	CellStar	188271	
Sterile dissecting forceps and scissors	N/A	N/A	
Sterile blades	Bard-Parker	371210	
Sterile petri dish	Fisher Scientific	FB012923	

## Materials and equipment

**Table:** Enzyme mix (Tumor dissociation, mouse kit, Miltenyi Biotec, Cat#130-096-730)

Reagent	Amount
DMEM	2.35 mL
Enzyme D	100 μL
Enzyme R	50 μL
Enzyme A	12.5 μL
**Total**	**2512.5** μL

**CRITICAL:** Thaw and keep enzyme mix on ice.

**Table:** DMEM +10%FBS

Reagent	Final concentration (mM or μM)	Amount
DMEM		44.5 mL
FBS	10%	5 mL
Penn/strep	1%	500 μL
**Total**		50 mL

***Note:*** Store at 2°C–8°C up to one month.

**Table:** DMEM +1%FBS

Reagent	Final concentration (mM or μM)	Amount
DMEM		9.8 mL
FBS	1%	100 μL
Penn/strep	1%	100 μL
**Total**		10 mL

***Note:*** Store at 2°C–8°C up to one month.

**Table:** 1**×** Binding Buffer (Dead cell removal kit)

Reagent	Final concentration (mM or μM)	Amount
20× Binding Buffer Stock	1**×**	500 μL
Sterile ddH_2_O		9.5 mL
**Total**		**10 mL**

***Note:*** Store at 2°C–8°C up to the date indicated by the manufacturer.

**Table:** 1**×** Red Blood Cell Lysis Solution

Reagent	Final concentration (mM or μM)	Amount
10× Red Blood Cell Lysis Solution	1**×**	1 mL
Sterile ddH_2_O		9 mL
**Total**		**10 mL**

***Note:*** Store the prepared solution at 18°C–25°C. Discard unused solution at the end of the day.

**Table:** Depletion Buffer (CD45+ depletion kit)

Reagent	Final concentration (mM or μM)	Amount
PBS (pH 7.2)	1**×**	49.650 mL
BSA	0.5%	250 μL
EDTA (1 M)	2mM	100 μL
**Total**		**50 mL**

***Note:*** Keep Buffer cold on wet ice (2°C–8°C). Discard unused solution at the end of the day.

## Step-by-step method details

### Tumor dissociation

**Timing: 1.5 h**

This step combines mechanical and enzymatic dissociation using gentleMACS Dissociator and gentleMACS Tubes. Gentle dissociation allows for obtaining a single-cell suspension with preserved cell characteristics for further single-cell RNA-Seq analysis.

1. Euthanize the mouse according to the IACUC guidelines.
2. Transcardially perfuse the animal with ice cold sterile PBS for 4–5 min or until the liver is cleared of blood.
3. Carefully excise pancreatic tumor with sterile dissecting tools without damaging nearby organs of gastro-intestinal tract to avoid bacterial contamination (Figure 1).

**Figure 1:**
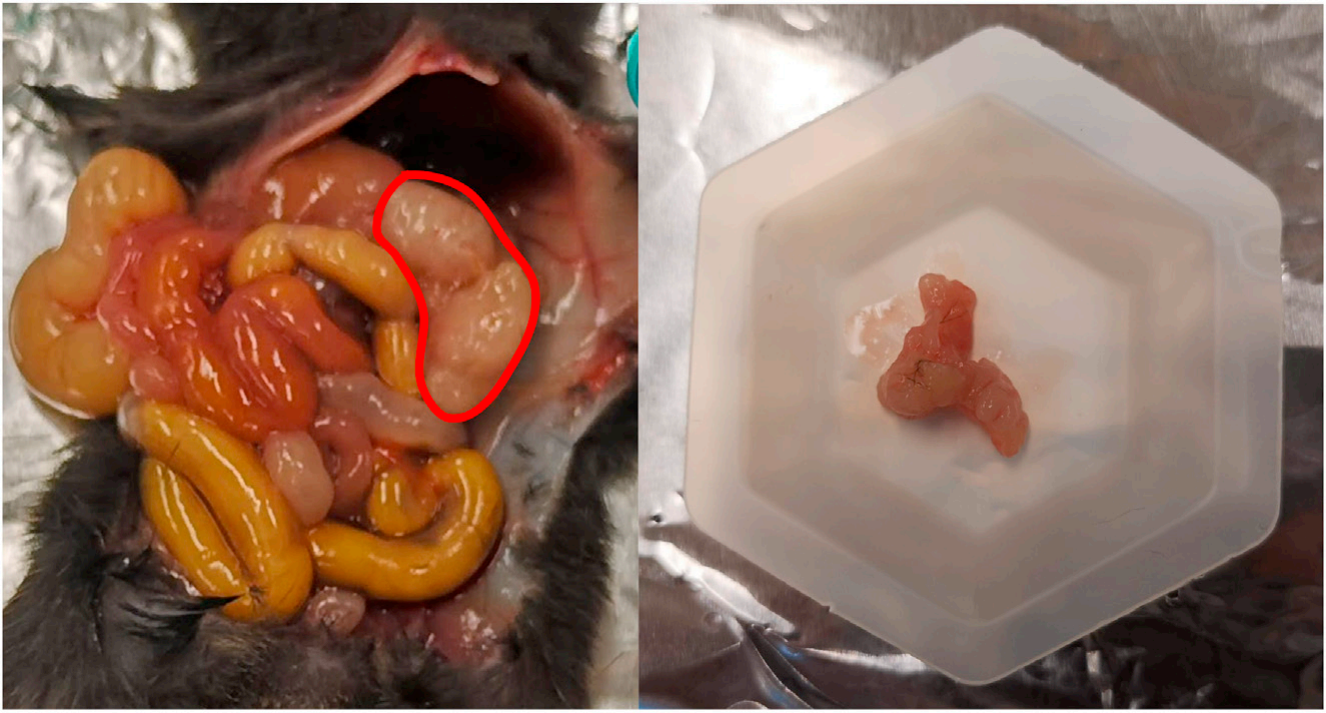
Excision of pancreatic tumor from the mouse The area of the tumor is outlined in red.
4. Wash the tumor with ice cold sterile PBS.
5. Remove fat and necrotic areas and excise blood vessels to the extent possible.

***Optional:*** Measure tissue weight if needed for further normalization of cell number per gram of tissue.

***Note:*** We recommend processing no more than two samples at a time.

6. Add 1 mL of enzyme mixture to the sterile petri dish and place the dish on ice.
7. Place tumor tissue in the dish with enzyme mix and cut to small pieces of 2–4 mm^3^ using sterile blades.

**CRITICAL:** Pieces have to be chopped finely to 1 mm or less.

**Figure 2:**
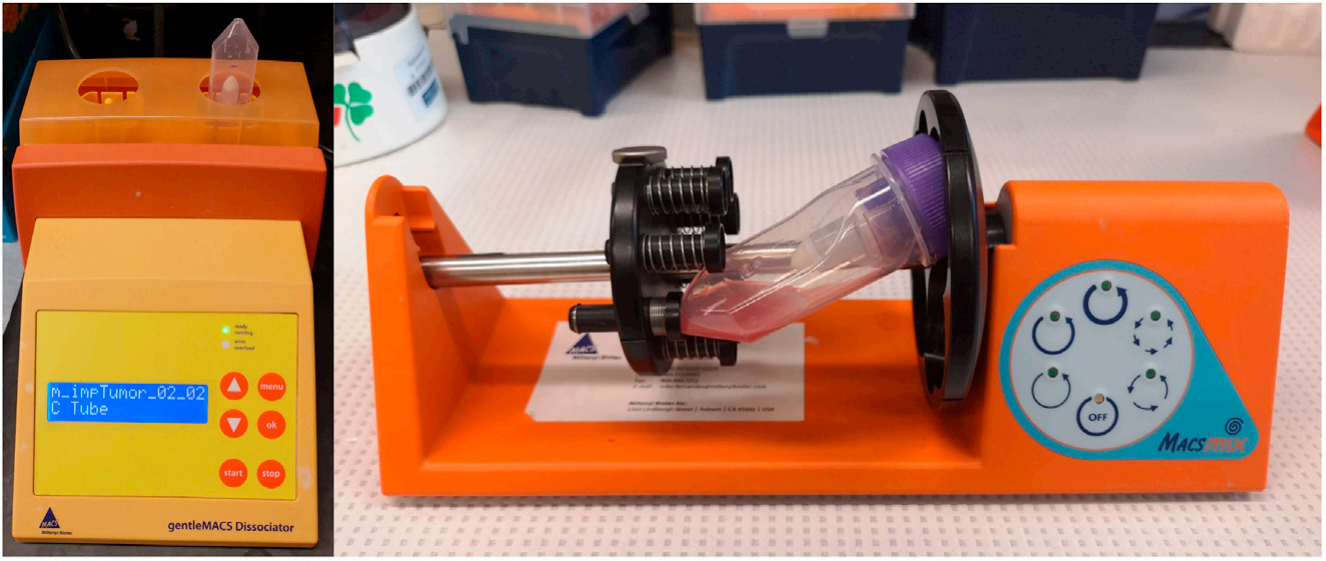
Tumor dissociation *Left,* C Tube placed on the gentleMACS Dissociator. *Right*, C tube placed on the MACSmix™ Tube Rotator.

8. Transfer tissue pieces into the gentleMACS C Tube containing the remaining enzyme mix.
9. Tightly close the C Tube, place it upside down onto the sleeve of the gentleMACS Dissociator at 18°C–25°C (Figure 2, *left*).

**CRITICAL:** Ensure that the sample material is located in the area of the rotor/stator.

10. Run program m_impTumor_02.

***Note:*** We recommend using predefined programs on gentleMACS Dissociator. However, testing different programs, or creating a custom program should aim at the best balance of viability versus yield of single-cell suspension.

11. Place the C Tube on the MACSmix™ Tube Rotator. Incubate for 40 min with continuous rotation at 37°C (Figure 2, *right*).
12. Place the C Tube on gentleMACS Dissociator, run program m_impTumor_03 twice (pre-defined on gentleMACS Dissociator).

**CRITICAL:** Ensure that the sample material is located in the area of the rotor/stator.

13. Perform a short (10 s) centrifugation to collect the sample material at the bottom of the tube.
14. Resuspend sample and apply the cell suspension to a 70 μm strainer placed on a 50 mL sterile falcon tube. Wash the strainer with 20 mL of RPMI-1640 or DMEM.
15. Apply the cell suspension to the new clean 70 μm strainer placed on a 50 mL sterile falcon tube one more time.

**CRITICAL:** Keep cells on ice from this point forward.

16. Determine total cell number in obtained single cell suspension manually using hemocytometer with Trypan Blue exclusion counting.

***Note:*** The starting amount of tumor tissue is usually not a limiting factor. You will need 700–1,200 cells/μl in the final solution. Cell viability should be more than 70% (preferably 90%). Purging apoptotic cells and/or CD45+ cells may significantly reduce the cell number. Apoptotic cell purging using Annexin V ferromagnetic beads is needed if viability is close or below 70%.

17. Centrifuge cell suspension at 300 *g* for 7 min at +4°C. Proceed immediately to dead cell removal.

### Dead cell removal

**Timing: 45 min to 1 h**

This step allows for reducing the amount of non-viable cells from a single cell suspension using Dead Cell Removal MicroBeads (Miltenyi Biotec). A large number of dead cells containing mitochondrial transcripts will be (by default) removed from the downstream analyses in Seurat or other platforms. Excessive loss of viability can affect the results of cell lineage profiling, of cells clustering, and interpretation of the experiment. When cell suspension passes through the column, the magnetically-labeled dead cells are retained within the column, and unlabeled living cells flow through.

**Figure 3:**
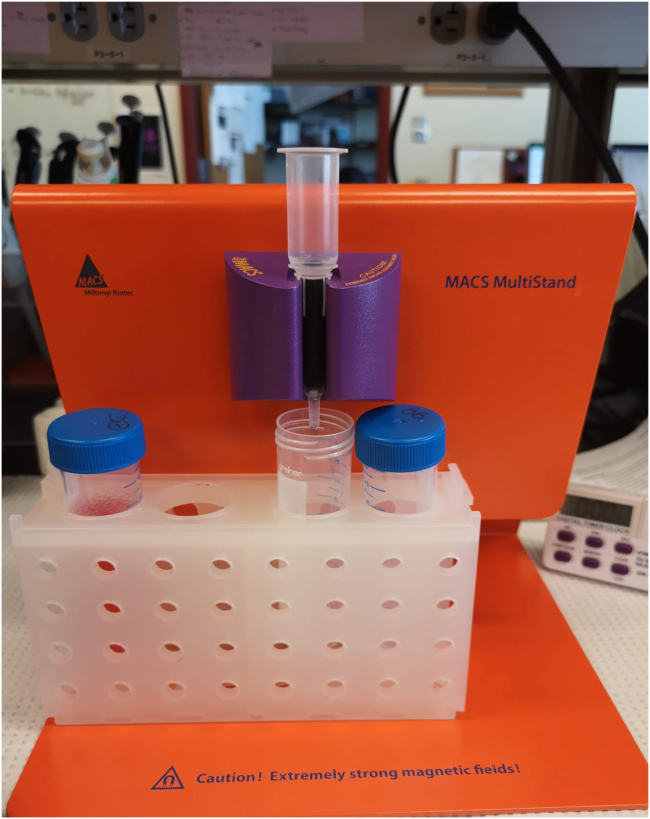
LS column placed in the MACS Separator
22. Apply cell suspension onto the column and collect flow-through containing unlabeled non-apoptotic cells (live cells).
23. Wash column with 1**×** Binding Buffer 4 times, adding 3 mL each time and collect all unlabeled cells that pass through combining it with the flow-through from step 21.

***Note:*** Always wait until the column reservoir is empty before proceeding to the next step.

18. Remove the supernatant.
19. Resuspend the cell pellet in 100 μL of Dead Cell Removal Microbeads per up to 10^7^ total cells. If cell number is higher than 10^7^ cells, scale up all reagent volumes accordingly. Mix well by pipetting and incubate for 15 min at 18°C–25°C.
20. While incubating, place the LS column in the magnetic field of the MACS Separator. Rinse the column with a 3 mL 1**×** Binding Buffer.
21. Place the 15 mL sterile falcon tube under the LS column (Figure 3).

24. Centrifuge the live cells at 300 *g* for 7 min at +4°C.
25. Remove the supernatant.

***Note:*** If there is a thick red layer in the cell pellet, proceed to Red Blood Cell Lysis followed by Leukocytes depletion. If the red layer is very thin or invisible, proceed directly to the Leukocytes depletion step.

***Optional:*** Mouse tissue perfusion via heart or portal vein cannulation with 10 mL of PBS helps to remove the red blood cells from the tissues.

### Red blood cell lysis (optional, but recommended if pellet has intense red color after the first centrifugation step)

**Timing: 15 min**

Red blood lysis provides lysis of erythrocytes in single-cell suspension with minimal effect on other cell types.

26. Resuspend the cell pellet in 10 volumes (10 **×** the pellet volume) of 1**×** Red Blood Cell Lysis Solution.
27. Vortex for 5 s and incubate the suspension for 2 min at 18°C–25°C.
28. Centrifuge the cells at 300 *g* for 10 min at 18°C–25°C.
29. Remove the supernatant and proceed to the next step.

### Leukocytes depletion

**Timing: 1 h**

CD45 MicroBeads are used in this step for the depletion of the leukocyte from the tumor tissue.

***Note:*** Do not use leukocyte depletion if immune cells are of interest.

***Note:*** All solutions need to be pre-cooled.

30. Determine total cell number manually or using automated hemocytometer.
31. Centrifuge at 300 *g* for 5 min at +4°C. Pipette off supernatant completely.
32. Resuspend the cell pellet in 90 μL of Depletion Buffer per 10^7^ total cells.
33. Mix well by pipetting and incubate for 15 min at +4°C
34. Wash cells by adding 1–2 mL of Buffer per 10^7^ cells and centrifuge at 300 *g* for 10 min at +4°C. Aspirate supernatant completely.
35. Resuspend the cell pellet in 500 μL of Buffer.

***Note:*** For higher cell numbers, scale up Buffer volume accordingly.

36. Place the LS column in the separator. Rinse the column with 3 mL of Buffer.
37. Apply cell suspension to the column to collect the flow through by gravity (leukocytes are trapped in the column).
38. Rinse the column with 3 mL 1**×** Buffer for 3 times. Collect all the flow-through in the collection tube and combine it with the flow-through from step 37.

***Note:*** These are unlabeled CD45-negative cells.

39. Remove the column from the separator and place it on a 15 mL tube.
40. Add 5 mL of Buffer to the column. Immediately flush out the magnetically labeled cells by firmly pushing the plunger into the column.

***Note:*** Always wait until the column reservoir is empty before proceeding to the next step.

41. Centrifuge the unlabeled cells at 300 *g* for 7 min at +4°C and proceed to the final preparation.

### Final preparation and counting

**Timing: 30 min**

42. Resuspend the cell pellet in 0.5 mL of DMEM+1%FBS.
43. Filter cell suspension with Flowmi ^TM^ Tip Strainers (70 μm) . Count cells using Trypan Blue and hemocytometer, observing viability and cell appearance (single, well separated cells without any cell conglomerates).
44. Repeat counting two times with two different aliquots.

**CRITICAL:** It’s critical to have well-separated single cells with viability no less than 90%. Dead cell removal and filtering should be repeated if needed.

45. Resuspend cells in DMEM+1%FBS for final concentration 700–1200 cells/μL with total cell number at least 7,000–12,000 cells.

***Note:*** The number of loaded cells is determined by the number of beads in each reagent kit which has to be matched close to 1:1 with the cell number. Gross deviation from the number recommended by the manufacturer's instructions would result in artifacts. For details on cell suspension volume calculation, refer to the manufacturer's manual (https://assets.ctfassets.net/an68im79xiti/4tjk4KvXzTWgTs8f3tvUjq/2259891d68c53693e753e1b45e42de2d/CG000183_ChromiumSingleCell3__v3_UG_Rev_C.pdf). We prefer manual counting in a hemocytometer with Trypan Blue exclusion staining.

### Generation of single-cell sequencing libraries

**Timing: 10 min**

46. Make a single-cell droplet with GEM beads using Chromium controller.
47. Convert single-cell suspension to barcoded scRNA-seq libraries by using the Chromium Single Cell 3′Library, Gel Bead & Multiplex Kit, and Chip Kit V3, loading an estimated 7,000–12,000 cells per library/well and following the manufacturer's instructions.
48. Carefully remove the tube from the Chromium Controller. It should appear milky. Without disturbing the emulsion, carefully place the tube(s) in a thermal cycler for reverse transcription.

**Pause point:** The sample can be left on the PCR machine until the next day.

**Figure 4:**
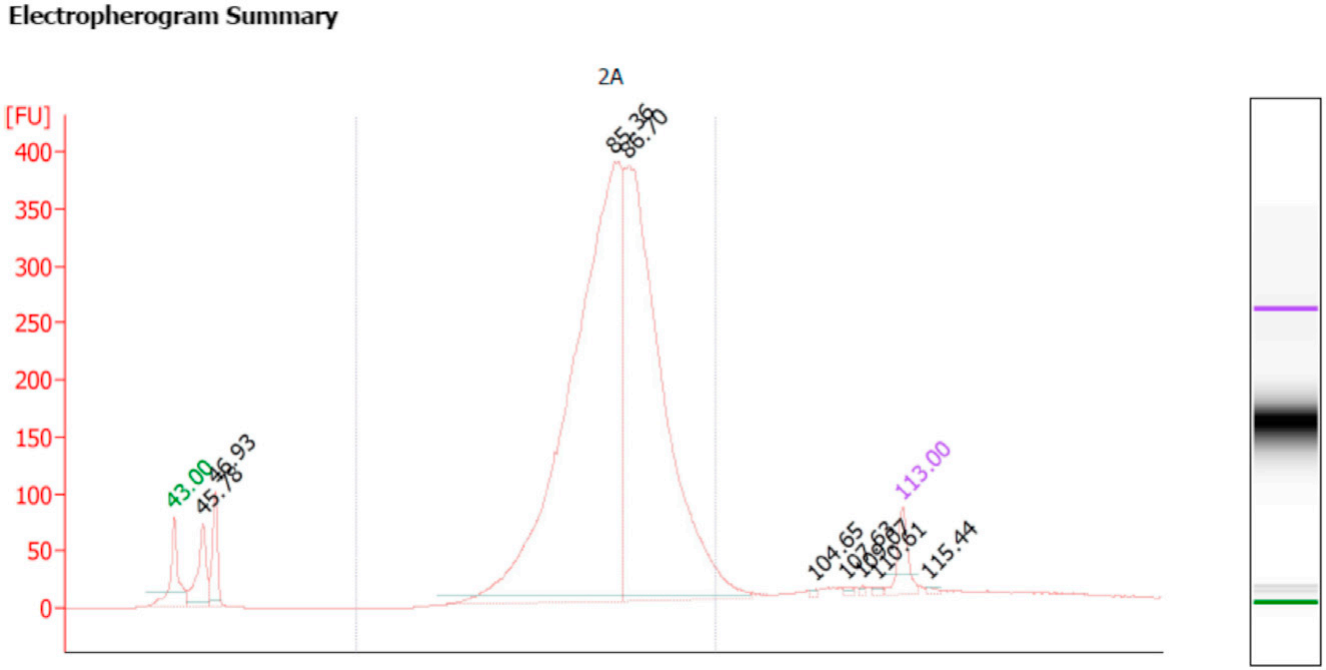
Example of a high-quality electropherogram of cDNA library The baseline is under 5 fluorescent units; a clearly identifiable single peak is well within the upper and lower bracket markers (upper marker- 10,380 bp, lower marker-35 bp); total cDNA amount is permitted within a relatively broad range of 1–1,900 ng. A ¼ (25%) fraction of the total cDNA is used for sequencing index PCR (SI-PCR). The yield of cDNA, however, defines the number of the SI-PCR cycles.

***Note:*** 10X Genomics recommends sequencing a minimum of 20,000 read pairs/cells for Single Cell 3′ v3/v3.1/HT v3.1/LT v3.1 and Single Cell 5′ v1.1/v2/HT v2 gene expression libraries. For Single Cell 3′ v2 libraries, we recommend 50,000 read pairs/cells.

49. Check the final libraries for cDNA quality using Bioanalyzer High Sensitivity DNA Kit, and quantify nucleic acids’ contents using the Qubit 2.0 (Figure 4). The total cDNA yield (ng) varies based on cell type, targeted cell recovery, etc. The number of secondary PCR cycles will depend on total cDNA yield. For the detailed information, refer to the manufacturer's manual (https://assets.ctfassets.net/an68im79xiti/4tjk4KvXzTWgTs8f3tvUjq/2259891d68c53693e753e1b45e42de2d/CG000183_ChromiumSingleCell3__v3_UG_Rev_C.pdf).

### Bioinformatics analysis of single-cell RNA sequencing

In the original paper (Gabitova-Cornell et al., 2020), analysis of scRNA-Seq data was conducted by manual command line and R processing. However, due to potential changes in tool versions, libraries and execution environments simply repeating the sequence of commands used in processing is likely to produce different results for different users (Sandve et al., 2013). In order to guarantee the reproducibility and portability of our analytic approach, we converted our analysis into reproducible Common Workflow Language (CWL) pipelines (https://github.com/common-workflow-language/cwltool/releases/tag/1.0.20170828135420) and executed them on user-friendly Scientific Data Analysis Platform (SciDAP, https://scidap.com). Open source CWL Pipelines used here are available at https://github.com/datirium/workflows. As a workflow runner we used CWL-Airflow (Kotliar et al., 2019), however, the same pipelines (https://doi.org/10.5281/zenodo.5339177 ) can be executed in any other CWL-based execution environments (https://commonwl.org).

**Figure 5:**
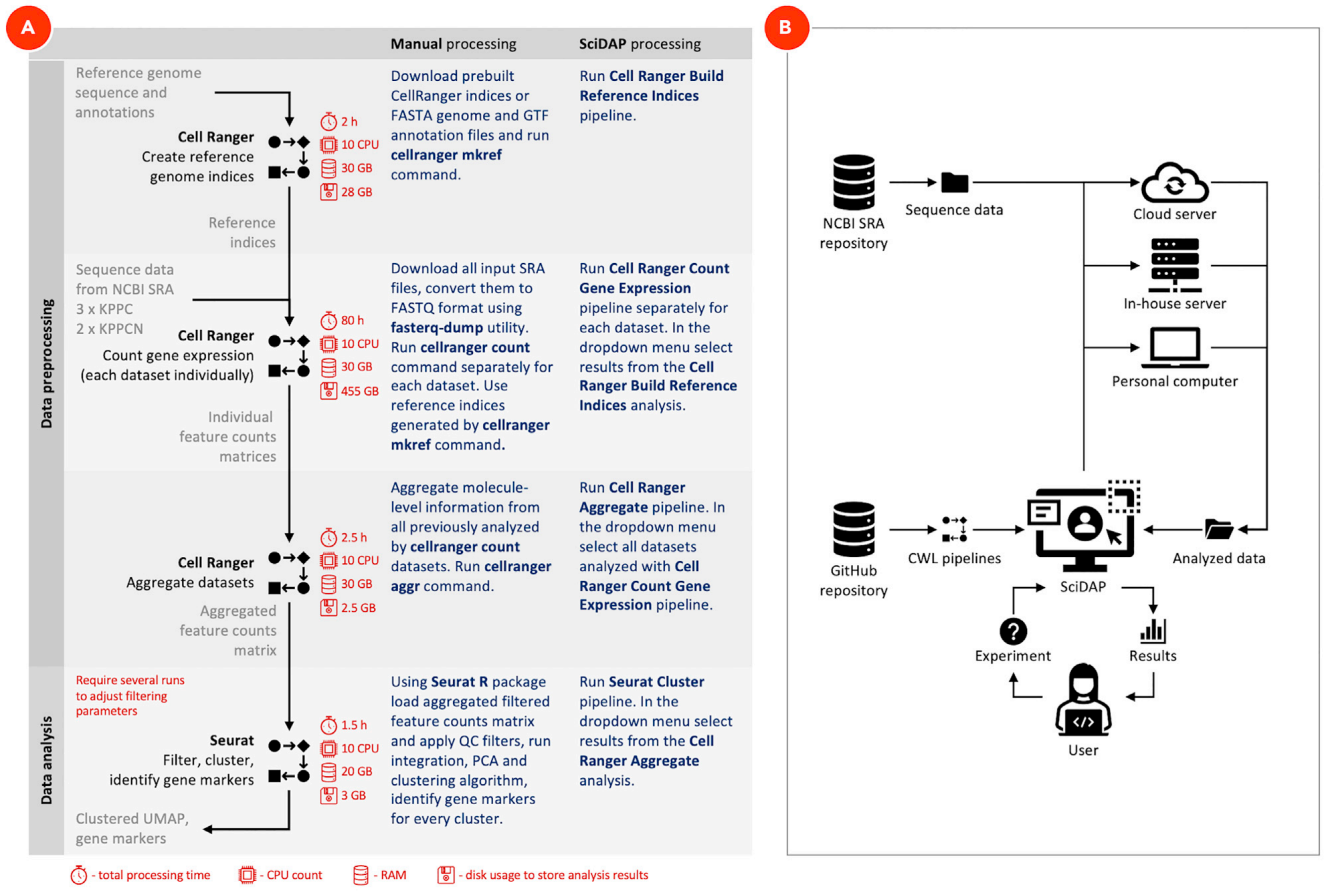
scRNA-Seq data analysis on SciDAP and by manual command line processing Timings calculated for the scRNA-Seq data analysis pipeline (A) show an actual processing time for each workflow run sequentially. All Cell Ranger based pipelines were limited both by 10 CPU and 30 GB of RAM. Seurat clustering analysis was limited only by 10 CPUs and at maximum used 20 GB of RAM. Disk usage to store data analyses results does not include the size of the input and temporary files. The preparation steps, such as downloading or uploading input data, installing and configuring required software are not included in timings. On the structural schema (B) all required pipelines are saved on GitHub repository following CWL specification that makes them portable and reproducible. After the user selects a workflow type, uploads or sets SRA run accession numbers for input data, the system automatically schedules workflow for execution on one of the available workers (personal computer, in-house or cloud server). When workflow finishes running, the user gets access to the data processing results.

CWL specification was developed (https://w3id.org/cwl/v1.0/) in order to make workflows portable and reproducible. It is a data-driven standard that describes tools and pipelines as YAML or JSON structured linked data documents. For each workflow step (“tool”), CWL defines inputs and outputs in a formalized way that allows for linking of tools into workflows. Moreover, CWL tool description includes a Docker (or Singularity) container supporting execution in an isolated runtime environment with a pre-installed and tested version of the required software. Therefore, the pipeline becomes independent of the execution platform and can be run in exactly the same way anywhere CWL standard is supported. A basic outline of scRNA-Seq pipeline as well as the structural schema of data processing on SciDAP is shown on Figure 5. The processing time for all Cell Ranger based workflows will depend on the quality of the input datasets, sequencing depth, number of detected cells, and available hardware resources. As for Seurat clustering pipeline, its performance will be mostly influenced by the input cells number, filtering thresholds and clustering resolution.

We recommend using specialized data analysis platform as it doesn’t require from a user having powerful computational resources. It also keeps data organized and guarantees workflows reproducibility as each workflow step is run inside a Docker container with the pre-installed and tested version of the required software. SciDAP provides a graphical user interface that allows biologists to analyze data using transparent, reproducible, and portable CWL pipelines without the need for coding. After the analysis is complete, biologists can explore results using interactive visualization tools and produce publication-ready images. Advanced users can adjust CWL pipelines and visualizations to suit their specific analysis needs. Below we will discuss how scRNA-Seq data analysis can be performed in SciDAP, how analysis parameters are chosen and briefly discuss what happens under the hood. The CWL pipelines used here are available on GitHub and can be used with any other CWL runner (see https://commonwl.org for full list), but without the graphical interface described here.

**Figure 6:**
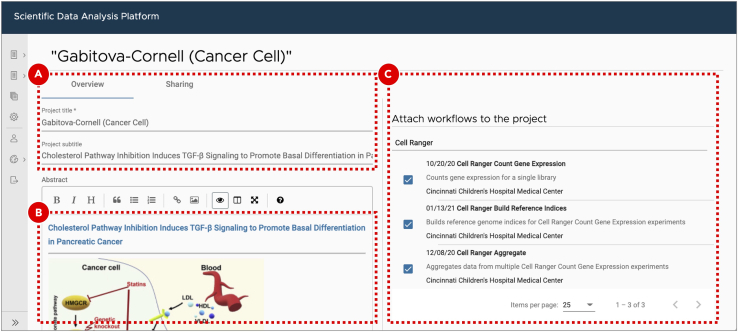
Creating a new project for scRNA-Seq data analysis Setting project title and subtitle helps to distinguish it from the other projects (A). A detailed project description can be added as a Markdown-formatted text (B). Since there are many ways to process the same data, only workflows that have been attached to the project can be used for data analyses to ensure that samples are directly comparable(C). The list of available workflows can be edited after project creation as well.
  d. Select the following workflows (Figure 6C) for single-cell RNA-Seq data analysis. For your convenience, use the search bar to find the required workflows faster.
    i. Cell Ranger Build Reference Indices
    ii. Cell Ranger Count Gene Expression
    iii. Cell Ranger Aggregate
    iv. Seurat Cluster
  e. Click the ***Save*** button at the top right corner of the screen.

**Figure 7:**
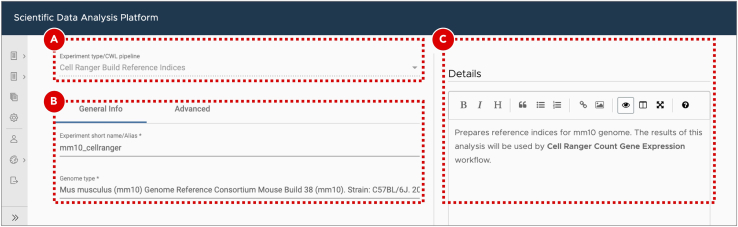
Building Cell Ranger reference indices After the user selects experiment type (A), they are presented with a form that allows them to specify experiment/analysis parameters (B). SciDAP automatically creates these input forms based on CWL pipelines. Optional experiment details can be added as a Markdown-formatted text (C).
  d. On the ***General info*** tab set an arbitrary ***Experiment short name*** to distinguish the newly created sample from the others. Select ***Mus Musculus (mm10)*** genome from the ***Genome type*** dropdown menu (Figure 7B). Optionally, add detailed experiment description in the ***Details*** section (Figure 7C). SciDAP already has the genome and annotation in its database and they will be used for indexing.
  e. Click the ***Save sample*** button at the bottom of the screen.
    **Pause point:** Before proceeding to the next step make sure that building Cell Ranger reference indices finished running successfully. If the analysis failed SciDAP displays an error marker with the percentage and the name of the last executed workflow step on the right side from the failed experiment on the ***Sample*** tab. SciDAP will not allow using failed experiments for further analyses.

**Figure 8:**
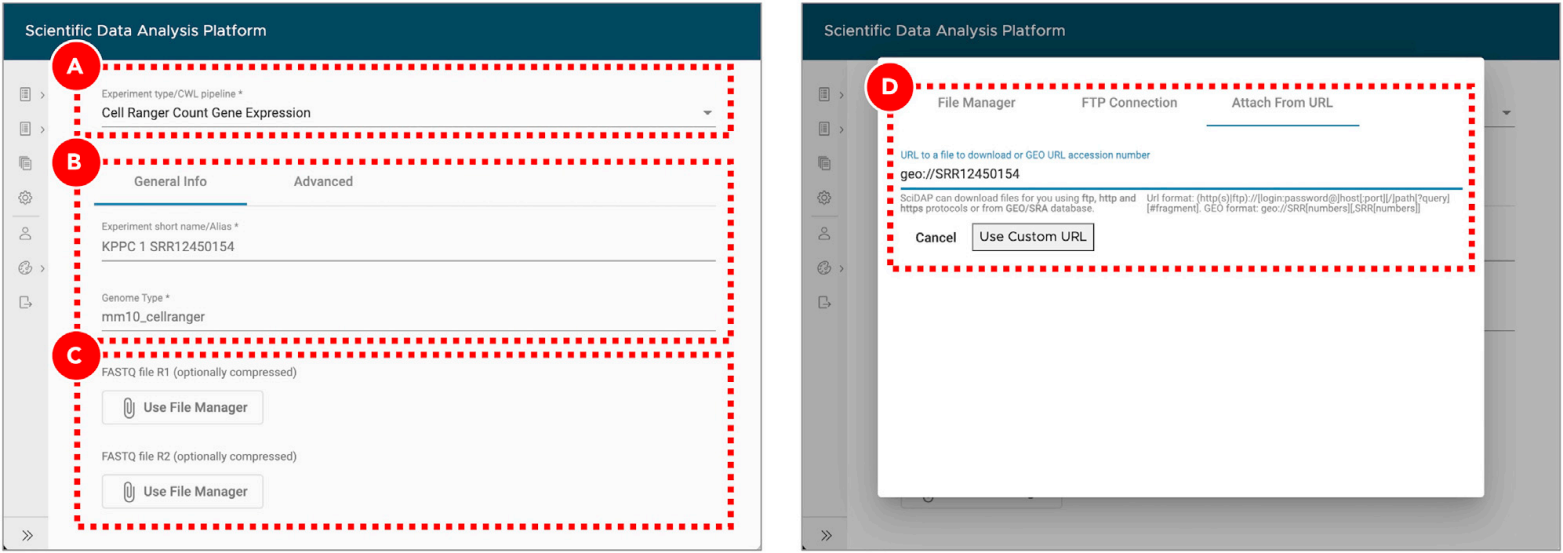
Gene expression quantification for KPPC 1 SRR12450154 dataset Experiment entry form allows users to select workflow type (A), set general parameters (B) and attach data files or provide accession number/URL to the data (C). For data from NCBI short read archive (SRA) SciDAP will automatically download and extract raw sequencing data into a pair of FASTQ files based on the SRR accession number (D). Same SRR number should be provided for both FASTQ R1 and R2 input files (C).
  d. On the ***General info*** tab set an arbitrary ***Experiment short name*** to distinguish the newly created sample from the others. Select the experiment with the Cell Ranger reference indices from the ***Genome type*** dropdown menu (Figure 8B). Optionally, add detailed experiment description in the ***Details*** section.
  e. Click the *Use File Manager* button under the *FASTQ file R1* label (Figure 8C).
  f. On the *Attach from URL* tab provide SRR run accession number in a form of geo://SRR12450154 (Figure 8D). Click the *Use custom URL* button.
  g. Provide the same SRR run accession number when clicking the *Use File Manager* button under the *FASTQ file R2* label.
  h. Click the *Save sample* button at the bottom of the screen. SciDAP will not allow adding failed or unfinished samples as input into ther next step. Users will be notified with the correspondent warning message about missing upstream analysis.
    ***Note:*** Repeat the same steps for each experiment to be analyzed (e.g. every SRX run in the **PRJNA657051** BioProject). The **KPPC 1 SRR12450154** dataset is shown as an example. To obtain a list of required SRR run accession numbers open https://www.ncbi.nlm.nih.gov/sra/?term=PRJNA657051 (Figure 9A) and copy SRR identifiers for each of the SRX experiments (Figure 9B).
    ***Optional:*** If sequence data from the multiple SRR runs belong to the same SRX experiment and should be processed as a single sample, SRR run accession numbers should be provided in a form of comma-separated list. In addition to downloading from NCBI SRA, ***Attach from URL*** tab also supports direct URLs to the FASTQ files deposited to other repositories.
    ***Alternatives:*** Input FASTQ files can be uploaded from the user’s computer using the ***File Manager*** tab or downloaded from the FTP server through the ***FTP Connection*** tab.
    The results of each successfully finished gene expression quantification experiment can be explored in a form of web-based report generated by Cell Ranger, and interactively in the UCSC Cell Browser (Speir et al., 2021). Additionally, a file compatible with the Loupe Browser (10X Genomics) can be downloaded from the ***Files*** tab. The user is presented with QC measures including the number of reads mapped to intergenic, exonic or intronic regions; the barcode rank plot and others.
    **Pause point:** Before proceeding to the next step make sure that gene expression quantification experiments successfully finished running for all five datasets.

50. Create a new project and attach workflows.
  In SciDAP, projects keep data organized by the study. Attaching workflows to projects ensures that similar data are processed by compatible pipelines.
  a. Log in or create a new user account on https://scidap.com. Follow the on-screen guides to set up your own laboratory or to join an existing one.
  b. Create a new project by clicking the ***New Project*** button on the ***Projects*** page.
  c. Set an arbitrary ***Project title*** and ***Project subtitle*** to distinguish the newly created project from the others (Figure 6A). Optionally, add detailed project description in the ***Abstract*** field below the ***Project subtitle*** section (Figure 6B).

51. Build Cell Ranger reference indices.
  To build reference genome indices Cell Ranger runs STAR (Dobin et al., 2013). Genome indices are used to make alignment algorithms fast and efficient. For Cell Ranger both genome sequences (FASTA) and gene annotation (GTF) files should be provided. The gene annotation file is required for splice junction extraction which improves mapping accuracy of scRNA-Seq data. More details about preparing genome references for Cell Ranger can be found on the official 10X Genomics documentation page (https://support.10xgenomics.com/single-cell-gene-expression/software/pipelines/latest/advanced/references).
  a. Enter a newly created project.
  b. Create a new experiment by clicking the ***Add sample*** button on the ***Sample*** tab.
  c. Select ***Cell Ranger Build Reference Indices*** workflow from the ***Experiment type*** dropdown menu (Figure 7A).

52. Quantify gene expression.
  Cell Ranger gene expression quantification starts with read trimming (for Single Cell 3′ Gene Expression) and running STAR for splice-aware read alignment. Only the reads that are uniquely mapped to the transcriptome are used for analysis. PCR duplicate reads are removed based on Unique Molecular Identifiers (UMI). Cell Ranger supports automatic sequencing error corrections in UMIs, which allows saving more reads. The unique reads that have valid cell barcodes and UMIs, and that are mapped to exactly one gene are used to create cell by gene matrix. More details about the Cell Ranger gene expression quantification algorithm can be found on the official 10X Genomics documentation page (https://support.10xgenomics.com/single-cell-gene-expression/software/pipelines/latest/algorithms/overview).
  a. Enter the project where the sample with the Cell Ranger reference indices was created.
  b. Create a new experiment by clicking the ***Add sample*** button on the ***Sample*** tab.
  c. Select ***Cell Ranger Count Gene Expression*** workflow from the ***Experiment type*** dropdown menu (Figure 8A).

**Figure 9:**
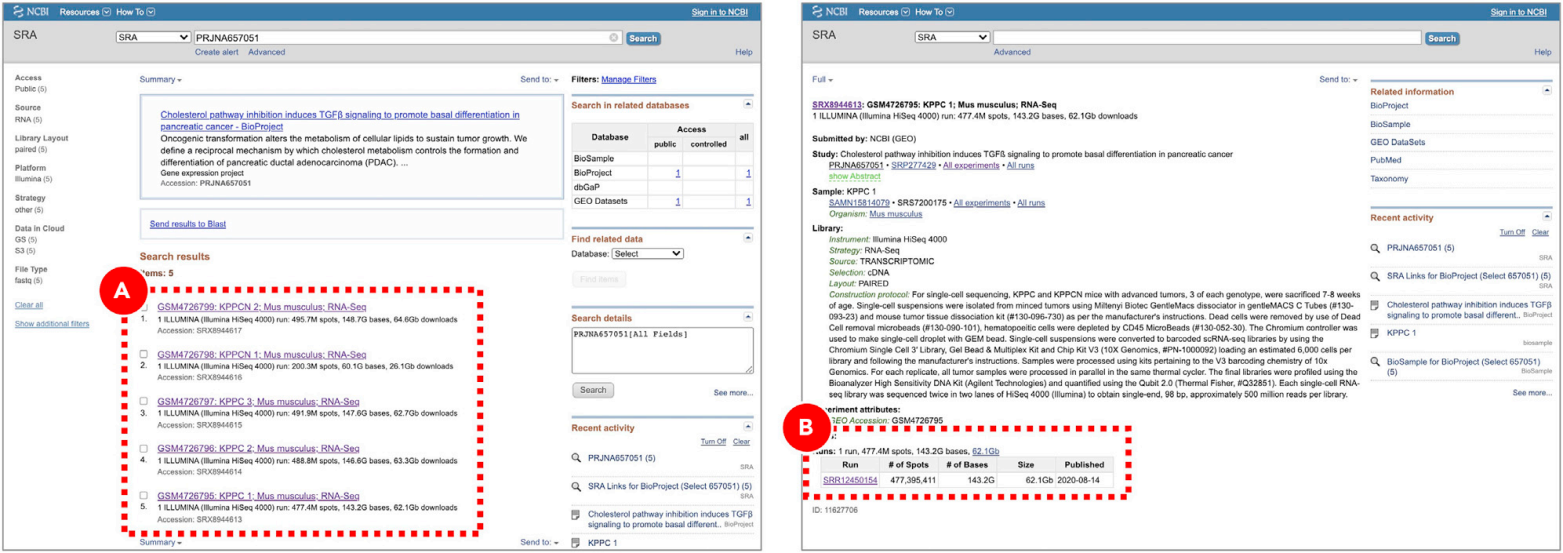
Searching for SRR run accession numbers that belong to the PRJNA657051 BioProject On NCBI SRA the raw sequencing data is saved in a form of SRA archives that can be accessed by their SRR run accession numbers (B). Multiple SRR runs can belong to the same SRX/GSM sample which in turn belongs to an SRP/GSE study (A). The same pages can be found via GEO search.

**Figure 10:**
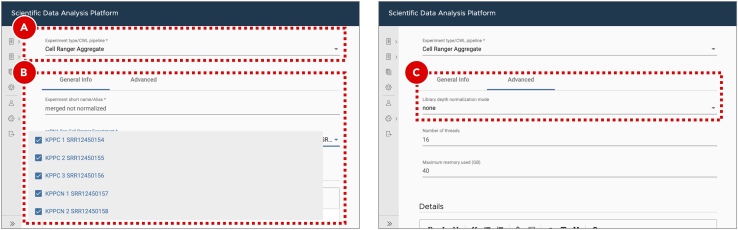
Aggregating gene expression from multiple datasets For Cell Ranger Aggregate workflow (A) setting library depth normalization to None (C) will disable the default behavior of normalizing the average read depth per cell between merged datasets (B). Normalization will be applied when integrating datasets with Seurat.
  d. On the ***General info*** tab set an arbitrary ***Experiment short name*** to distinguish the newly created sample from the others. Select all five Cell Ranger Count Gene Expression experiments from the ***scRNA-Seq Cell Ranger Experiment*** dropdown menu (Figure 10B). Optionally, add detailed experiment description in the ***Details*** section.
  e. On the ***Advanced*** tab set ***Library depth normalization mode*** to ***None*** (Figure 10C).
  f. Click the ***Save sample*** button at the bottom of the screen.
    ***Optional:*** The results of successfully finished gene expression aggregation experiment can be explored in a form of web-based report generated by Cell Ranger, and interactively in the UCSC Cell Browser (Speir et al., 2021). Additionally, a file compatible with the Loupe Browser (10X Genomics) can be downloaded from the ***Files*** tab.
    **Pause point:** Before proceeding to the next step make sure that gene expression aggregation experiment finished running successfully

**Figure 11:**
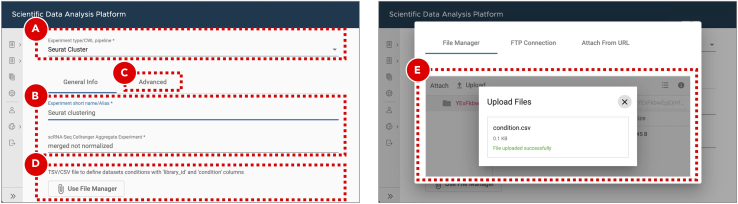
Clustering and gene markers identification For Seurat Cluster workflow (A) the file defining sample groups can be uploaded through the experiment entry form (D and E). In case it is not provided each dataset from the selected scRNA-Seq Cell Ranger Aggregate Experiment (B) will be assigned to its own separate group. The default filtering and clustering parameters can be adjusted on the Advanced tab (C).
  d. On the ***General info*** tab set an arbitrary ***Experiment short name*** to distinguish the newly created sample from the others. Select Cell Ranger Aggregate experiment from the ***scRNA-Seq Cell Ranger Aggregate Experiment*** dropdown menu (Figure 11B). Optionally, add detailed experiment description in the ***Details*** section.
  e. On the ***Advanced*** tab (Figure 11C) set workflow execution parameters listed in the **Adjusted** column of Table 2. The logic behind updating the default clustering parameters is explained in the **Explore clustering results** section. With a new dataset, we recommend to first perform analysis using the default parameters and then based on QC results adjust the parameters and repeat the Seurat analysis.

53. Aggregate cells by gene data from multiple samples.
  To proceed to the clustering analysis, the results of all five gene expression quantification experiments should be merged into a single feature-barcode matrix. However, since for each scRNA-Seq experiment, cell barcodes were drawn from the same pool of whitelisted barcodes (10X Genomics) a simple merging may result in having duplicated barcodes. To avoid this scenario Cell Ranger updates each barcode with an integer suffix pointing to the dataset the cell came from before running aggregation. Optionally, Cell Ranger may run a depth normalization algorithm to make all merged datasets have a similar number of uniquely mapped to transcriptome reads per cell. This approach may be suboptimal since all data will be downsampled to match the worst sample. Here we aggregate samples without normalization, leaving normalization to Seurat. More details about the Cell Ranger aggregation algorithm can be found on the official 10X Genomics documentation page (https://support.10xgenomics.com/single-cell-gene-expression/software/pipelines/latest/using/aggregate).
  a. Enter the project where the sample with the Cell Ranger reference indices was created.
  b. Create a new experiment by clicking the ***Add analysis*** button on the ***Analysis*** tab.
  c. Select ***Cell Ranger Aggregate*** workflow from the ***Experiment type*** dropdown menu (Figure 10A).

54. Cluster and identify gene markers.
  The joint analysis of multiple scRNA-Seq datasets with Seurat (Hao et al., 2021) starts with evaluation of common single-cell quality control (QC) metrics – genes and UMIs counts, percentage of mitochondrial genes expressed. QC allows to get a general overview of the dataset quality as well as to define filtering thresholds for dead or low-quality cell removal. Filtered merged datasets are then being processed with the integration algorithm. Its main goal is to identify integration anchors – pairs of cells that can “pull together” the same cell type populations from the different datasets. An integration algorithm can also solve batch correction problems by regressing out the unwanted sources of variation. The integrated data then undergo the dimensionality reduction processing that starts from the principal component analysis (PCA). Based on the PCA results the uniform manifold approximation and projection (UMAP) and clustering analysis are run with the principal components of the highest variance. Clustered data are then used for gene markers identification. These genes are differentially expressed between clusters and can be used for cell types assignment. More details about scRNA-Seq integration analysis with Seurat can be found on the official documentation page (https://satijalab.org/seurat).
  a. Enter the project where the sample with the Cell Ranger reference indices was created.
  b. Create a new experiment by clicking the ***Add analysis*** button on the ***Analysis*** tab.
  c. Select ***Seurat Cluster*** workflow from the ***Experiment type*** dropdown menu (Figure 11A).

**Table 2:** Advanced filtering, clustering and gene markers identification parameters

Group	Parameter	Default	Adjusted	Comment
QC filtering	Include genes detected in at least this many cells	10	10	
	Include cells where at least this many genes are detected	250	300	Adjusted to remove outliers based on the genes per cell density distribution violin and ranked cells plots.
	Include cells with the number of genes not bigger than this value	5000	6200	
	Include cells where at least this many UMIs are detected	500	500	
	Include cells with the novelty score (the ratio of genes per cell over UMIs per cell) not lower than this value	0.8	0.8	
	Include cells with the percentage of transcripts mapped to mitochondrial genes not bigger than this value	5	5	
	Pattern to identify mitochondrial genes	ˆMt-	ˆmt-	Mitochondrial genes from the mouse genome start with lowercase “mt-“.
Integration and dimensional reduction	Number of highly variable genes to detect (used for dataset integration and dimensional reduction)	3000	3000	
	Number of principal components to use in UMAP projection and clustering (from 1 to 50)	10	20	Adjusted to point to PC20 where the curve on the Elbow plot starts to plateau
	Regress cell cycle as a confounding source of variation	False	False	
	Regress mitochondrial gene expression as a confounding source of variation	False	False	
	Clustering resolution	0.1	0.5	
Gene markers identification	Include only those genes that on average have log fold change difference in expression between every tested pair of clusters not lower than this value	0.25	0.25	
	Include only those genes that are detected in not lower than this fraction of cells in either of the two tested clusters	0.1	0.1	
	Statistical test to use for gene markers identification	Wilcox	Wilcox	
	Report only positive gene markers	False	True	Uses only genes that are overexpressed in a given cluster as putative markers. If False, both overexpressed and silent genes will be reported.
Gene expression	Comma or space separated list of genes of interest	None	*Clec3b, Il6, Lgals7, Pdgfra, Vim, Tgfb1, Ptprc, Epcam, Cldn4, Krt7, Sox9, Cdh1, Upk3b, Mki67*	Genes of biological interest can be selected for plotting.
  f. In a text editor or Microsoft Excel create comma-separated ***condition.csv*** file to assign each dataset to either KPPC or KPCCN group (Table 3). ***library_id*** column includes values used in ***Experiment short name*** fields of Cell Ranger Count Gene Expression experiments, ***condition*** column defines the group to which each dataset should be assigned.

**Table 3:** Comma-separated *condition.csv* file to define datasets grouping

library_id	Condition
KPPC 1 SRR12450154	KPPC
KPPC 2 SRR12450155	KPPC
KPPC 3 SRR12450156	KPPC
KPPCN 1 SRR12450157	KPPCN
KPPCN 2 SRR12450158	KPPCN
  g. Click the ***Use File Manager*** button (Figure 11D) to upload the newly created ***condition.csv*** file.
  h. On the ***File Manager*** tab click the ***Upload*** button and find the previously saved ***condition.csv*** file. Once the file is successfully uploaded select it from the list and click the ***Attach*** button (Figure 11E).
  i. Click the ***Save sample*** button at the bottom of the screen.
    **Pause point:** Before proceeding to the next step make sure that clustering and gene markers identification experiment finished running successfully.
    ***Optional:*** Explore clustering results
    Cell Ranger Count Gene Expression pipeline uses an advanced cell-calling algorithm that allows the identification of high-quality cells from each dataset. It also runs a preliminary clustering analysis that may be sufficient for some cases. However, in addition to that, we recommend exploring commonly used QC metrics for the merged datasets that are produced by Seurat. These will help to evaluate how filtering parameters influence the number of remaining cells, define datasets dimensionality, perform clustering, identify gene markers, and evaluate expression levels of genes of interest to assign cell types to clusters. All of these are accessible on the ***QC (not filtered)***, ***QC (filtered)***, ***Dimensionality evaluation***, ***QC (integrated)***, ***Clustering***, ***Gene expression***, and ***Putative gene markers*** tabs of the successfully finished clustering experiment.
  j. Cell count bar plot (Figure 12A) allows to examine the number of cells per dataset. Cell counts mainly depend on the number of loaded cells and on the capture efficiency of the used single-cell protocol. The minimum required number of cells can be estimated based on the assumed number of cell types, the minimum fraction of the rarest cell type in population, and the minimum required number of cells per type (https://satijalab.org/howmanycells/).

**Figure 12:**
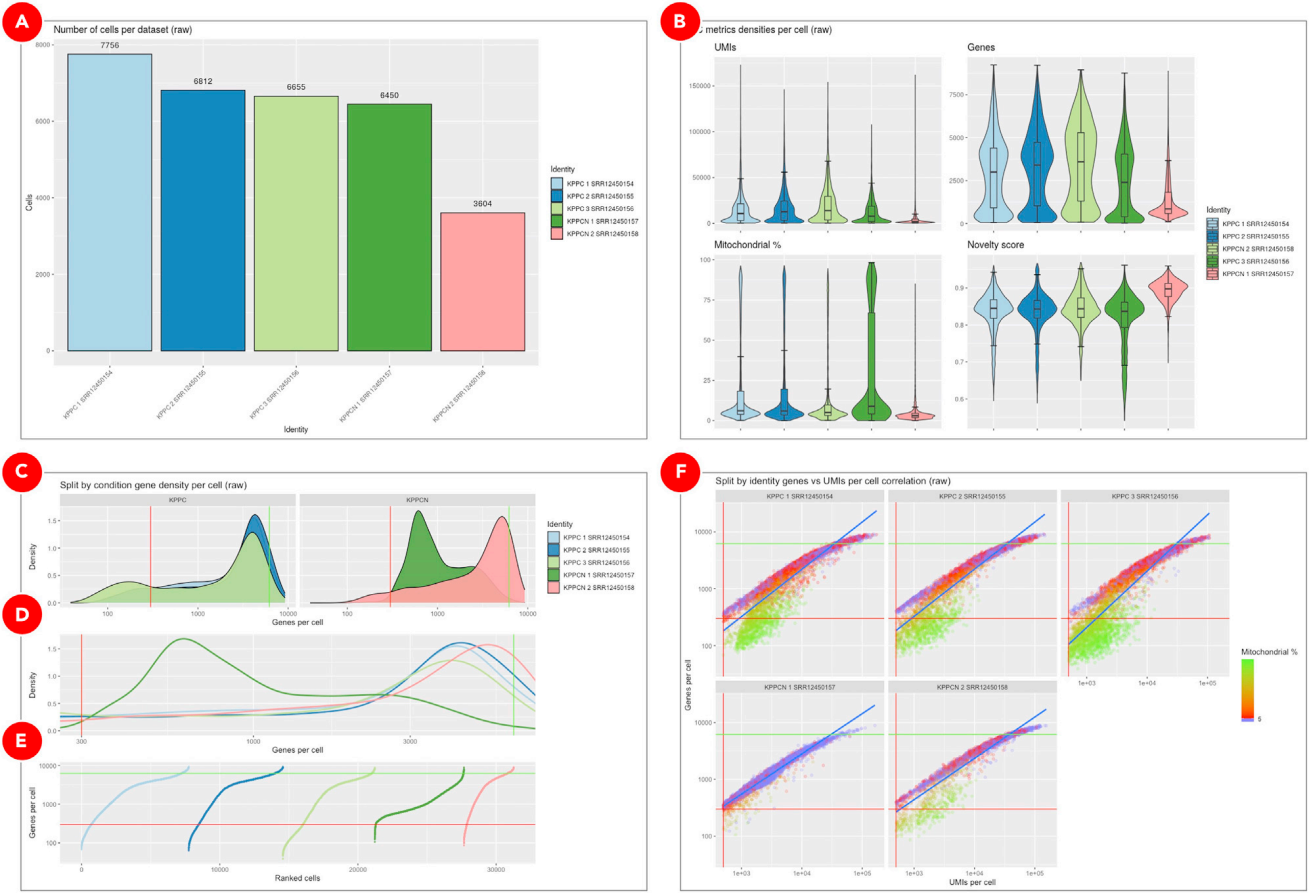
QC metrics for merged datasets Cell count bar plot (A) shows the number of cells per dataset. The violin plots (B) are used to visualize density distributions of the main QC metrics. Genes per cell density distribution plot (C) is split into KPPC and KPPCN groups. Zoomed in section of the density plot (D) displays all 5 datasets within the selected boundaries. Cell rank plot (E) displays cells sorted by gene per cell counts within each dataset. The lower and upper limits for genes per cell values are shown as red and green lines correspondingly. On the genes per cell over UMIs per cell correlation plot (F) a vertical red line indicates the minimum threshold for UMIs per cell values. All the cells with the percentage of transcripts mapped to mitochondrial genes below 5% are marked as blue.
  k. The violin plots (Figure 12B) are used to visualize per cell density distributions of the following metrics: UMI counts, gene counts, the percentage of transcripts mapped to mitochondrial genes, and novelty score. Embedded box plots help to spot outliers and define filtering thresholds for each metrics. In general, it is recommended to filter out all of the cells with less than 500 UMIs per cell. The abundance of cells with low UMIs per cell values may indicate a small number of reads uniquely mapped to transcriptome or low sequencing depth. The percentage of transcripts mapped to mitochondrial genes correlates with the number of dying cells. In most of the cases cells with more than 5% of the reads mapped to mitochondrial genes should be discarded. The novelty score indicates the complexity of the dataset and is calculated as the ratio of genes per cell over UMIs per cell. Typically, the novelty score should be above 0.8. Higher novelty score implies higher diversity of genes per UMIs and requires more cells to be called per dataset.
  l. To evaluate the lower and upper limits for gene per cell counts not only the density distribution (Figure 12B–12D), but also the cell rank plot (Figure 12E) can be used. The latter allows to visually identify the cells with extremely low or extremely high number of genes for each dataset. In general, to filter out low-quality cells or empty droplets the minimum threshold for gene per cell counts should be set to 500. Setting the upper limit for these metrics can be used to remove cell doublets.
  m. All three metrics combined (UMI counts, gene counts, the percentage of transcripts mapped to mitochondrial genes) are shown on the genes per cell over UMIs per cell correlation plot (Figure 12F). This plot can be used to evaluate whether a certain threshold has already discarded all unnecessary cells, so the other filtering criteria can be omitted, thus avoiding accidental removal of viable cell populations.
  n. A combined effect of filtering by UMI counts, gene counts, and by the percentage of mitochondrial reads is shown on the genes per cell over UMIs per cell correlation plot (Figure 13A). The plot displays the remaining cells after all QC filters have been applied.

**Figure 13:**
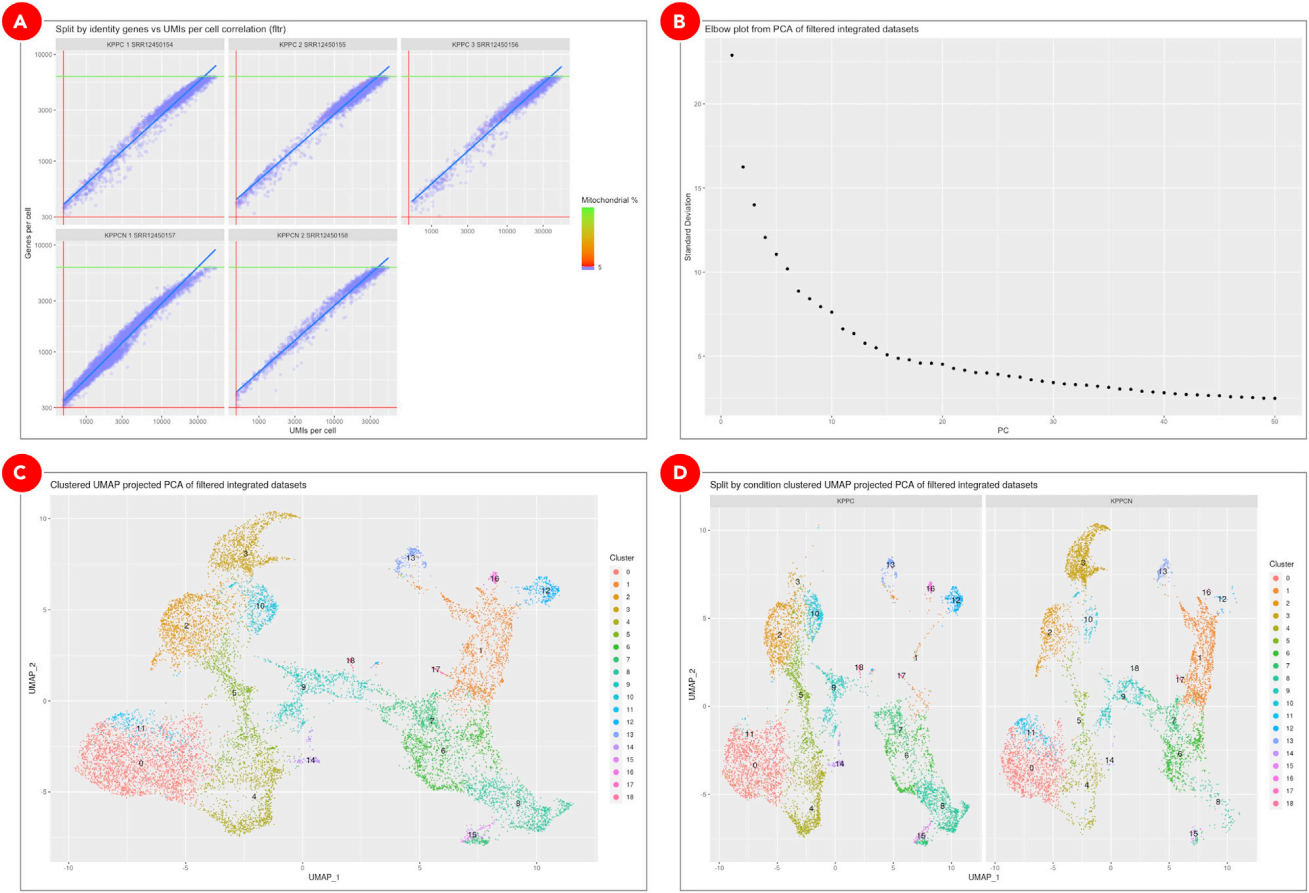
QC metrics and dimensional reduction analysis of filtered integrated datasets Combined effect of multiple filtering parameters can be explored on the genes per cell over UMIs per cell correlation plot (A). Elbow plot (B) helps to define datasets dimensionality, but it is rather a subjective measure and should not be used as the main criteria. Final clustering results (C) as well as split by condition clusters (D) are used to evaluate cell populations present in datasets.
  o. The Elbow plot (Figure 13B) is used to evaluate the dimensionality of the filtered integrated datasets by selecting only those principal components that capture the majority of the data variation. Typically, it is defined by the principal component after which the plot starts to plateau.
  p. UMAP plot shows cell clusters in the filtered integrated dataset (Figure 13C). The same plot but split into KPPC and KPPCN groups (Figure 13D) allows to spot clusters distinctive for each group.
  q. Dot plot with scaled gene expression (Figure 14A) is used to visually evaluate the average expression levels and the percentage of genes of interest per cluster. It helps to explore the similarities between clusters and identify cell types.

**Figure 14:**
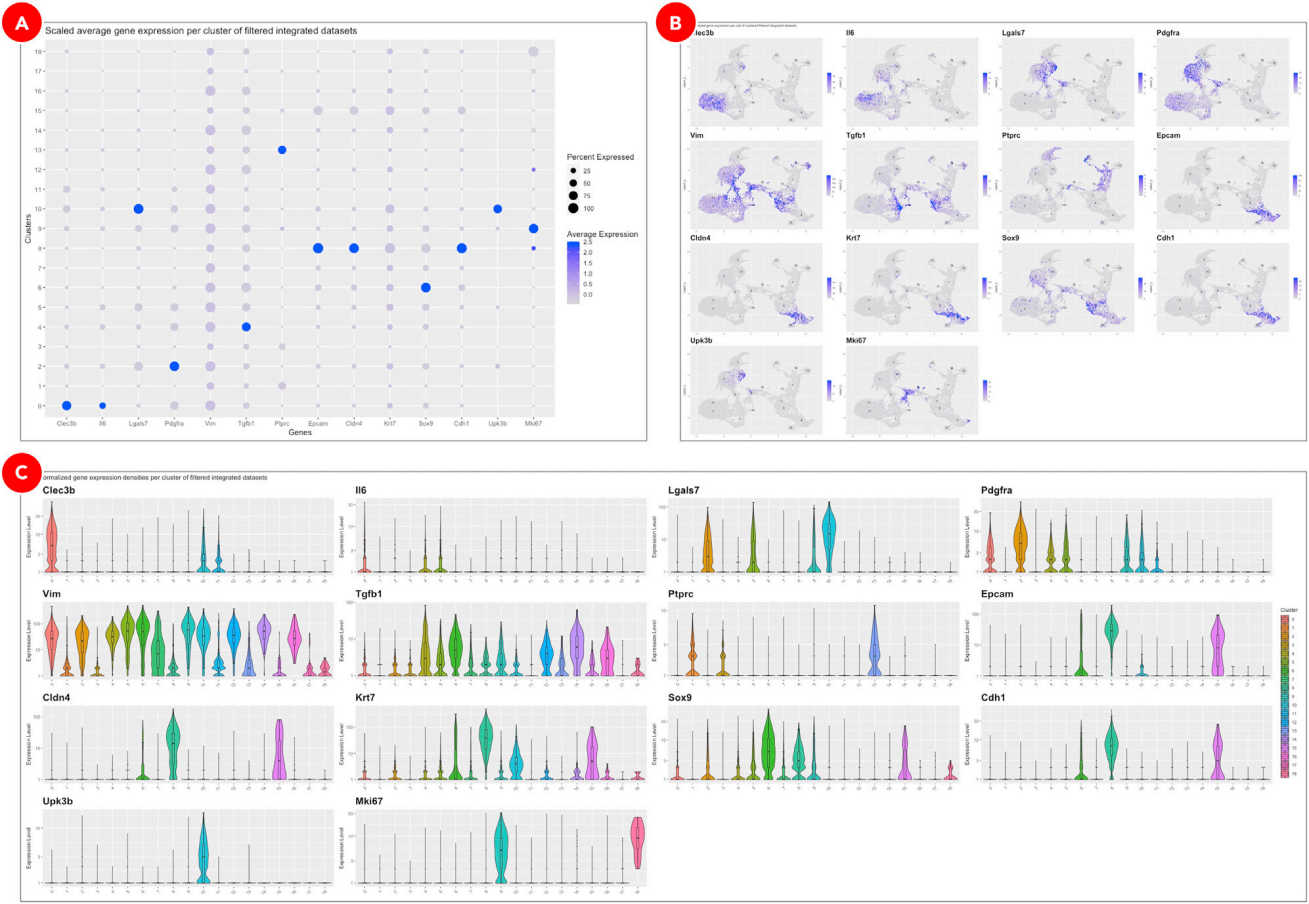
Gene expression plots for assigning cell types to clusters Clusters on a dot plot (A) are grouped together based on the similarities of the expression levels of genes of interest. On the feature plots (B) cells are highlighted correspondingly to the normalized expression levels of each of the genes of interest. Violin plots (C) show gene expression for genes of interest in each cluster.
  r. Feature plots (Figure 14B) highlight normalized expression levels of genes of interest over the identified clusters.
  s. The violin plots (Figure 14C) show the density distributions of the normalized expression levels of genes of interest per cluster. Altogether with above mentioned plots they are used for manual assigning cell types to clusters.
  t. Clustering results loaded in UCSC Cell Browser allow to interactively explore identified clusters (Figure 15A), highlight cells belonging to various samples and conditions (Figure 15B), explore expression of genes of interest (Figure 15C), and export barcodes of selected cells (Figure 15D) for further analyses.

**Figure 15:**
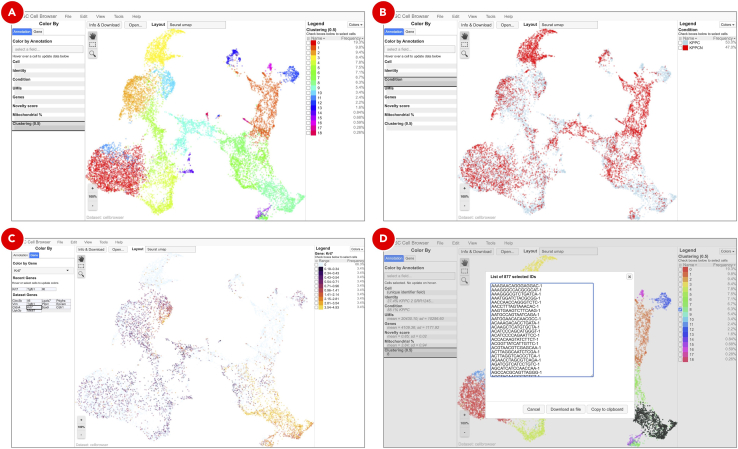
Clustering results visualized in UCSC Cell Browser (next page) Depending on the option selected on the Annotation tab, UCSC Cell Browser highlights identified clusters (A), groups datasets by specified condition (B), colors cells based on the expression of genes of interest (C), and generates a barcodes list for a selected group of cells (D).
  u. On the ***Putative gene markers*** tab (Figure 16A) an interactive table includes gene markers for each cluster. The column names correspond to the output of ***FindAllMarkers*** function (Seurat 4.0.1 R package). On the ***Files*** tab (Figure 16B) the list of all generated files is available for download. Among these files the ***seurat_clst_data_rds.rds*** (Figure 16C) includes Seurat clustering data in a format compatible with RStudio.

**Figure 16:**
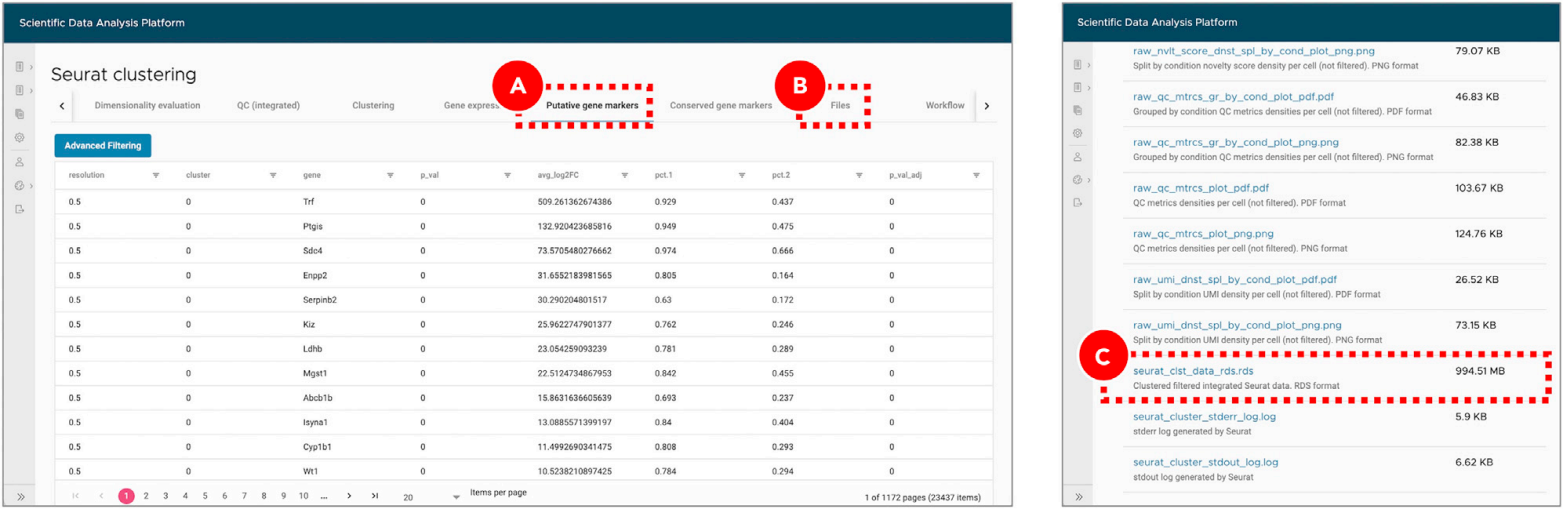
Gene markers identification and direct download of workflow execution results In the putative gene markers table (A), the values in *avg_log2FC* column are calculated as a log2 fold change difference between the average gene expression in the current cluster compared to all other clusters combined. The *pct.1* and *pct.2* columns show the percentage of cells with the specific gene expressed in the current cluster and all other clusters combined. This table can be used to identify gene markers of interest and assign cell types. Seurat clustering results can be downloaded in a compatible with RStudio format (C) from the *Files* tab (B).

## Expected outcomes

This protocol provides the reproducible method for mouse pancreatic tumor dissociation and preparation of single-cell suspension for single-cell RNA sequencing analysis. The protocol was used for mice with advanced tumors at 7–8 weeks of age. After mechanical and enzymatic digestion, dead cells removal, red blood cell lysis, and leukocytes’ depletion the final single-cell suspension consisted of populations of tumor cells and stromal non-myeloid cells. The data analysis part of protocols allows to identify cell populations that are common and distinct between cancer types.

## Limitations

**Figure 17:**
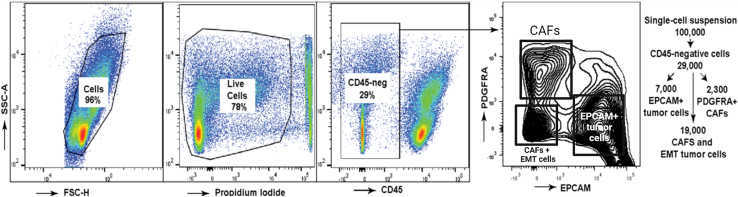
Flow cytometry analysis of a representative single-cell suspension isolated from a mouse pancreatic tumor Estimated number of cell types based on single-cell RNA sequencing data is shown on the right panel.

Because pancreatic tumors contain significant percentage of inflammatory hematopoietic cells (nearly ½ of all cellular population isolated using the enzymatic digestion procedures described above (Dominguez et al., 2020)), and because of the limited cell number allotted for creating single-cell RNA expression libraries, removal of CD45-expressing cells may be necessary for the applications when the investigators are interested in capturing the minority cellular populations such as specific subsets of epithelial cancer cells or cancer-associated fibroblasts (Peng et al., 2019). We recommend flow cytometry analysis of the isolated cell populations to enrich the desired cell types if necessary (Figure 17).

## Troubleshooting

### Problem 1

Poor viability of cells following enzymatic digestion.

### Potential solution

We found significant batch and vendor variability of the enzymatic preparations which could affect the viability of cells at the end of enzymatic digestion. Test the amount of enzyme and titrate the dilutions to achieve the optimal balance of viability and tissue disintegration for single cell isolation.

### Problem 2

Low number of the desired cell population.

### Potential solution

Consider negative or positive selection enrichment strategies for the desired cell population based on the established surface markers applicable to live cells. Refer to vendors for available reagents: https://www.miltenyibiotec.com/ or others.

We and others (Dominguez et al., 2020; Francescone et al., 2021) found that fibroblastic cells are more difficult to isolate from fibrotic tumor samples. Longer enzymatic digestion may be needed.

## Resource availability

### Lead contact

Further information and requests for resources and reagents should be directed to and will be fulfilled by the lead contact, Igor Astsaturov, Fox Chase Cancer Center, 333 Cottman Avenue, Philadelphia, PA 19111. Phone (215) 214–4297; Fax (215) 728–3639, e-mail: igor.astsaturov@fccc.edu

### Materials availability

This study did not generate a new unique reagent.

## Data Availability

The datasets generated during this study are available at Sequence Read Archive (SRA), https://www.ncbi.nlm.nih.gov/sra, deposition PRJNA530747. Source code for CWL pipelines is available from https://github.com/datirium/workflows
